# Genetic regulation and function of epidermal growth factor receptor signalling in patterning of the embryonic *Drosophila* brain

**DOI:** 10.1098/rsob.160202

**Published:** 2016-12-14

**Authors:** David Jussen, Janina von Hilchen, Rolf Urbach

**Affiliations:** Institute of Genetics, University of Mainz, 55099 Mainz, Germany

**Keywords:** dorsoventral patterning genes, epidermal growth factor receptor, *rhomboid*, *vein*, *argos*, brain neuroblasts

## Abstract

The specification of distinct neural cell types in central nervous system development crucially depends on positional cues conferred to neural stem cells in the neuroectoderm. Here, we investigate the regulation and function of the epidermal growth factor receptor (EGFR) signalling pathway in early development of the *Drosophila* brain. We find that localized EGFR signalling in the brain neuroectoderm relies on a neuromere-specific deployment of activating (Spitz, Vein) and inhibiting (Argos) ligands. Activated EGFR controls the spatially restricted expression of all dorsoventral (DV) patterning genes in a gene- and neuromere-specific manner. Further, we reveal a novel role of DV genes—*ventral nervous system defective* (*vnd*), *intermediate neuroblast defective (ind), Nkx6*—in regulating the expression of *vein* and *argos*, which feed back on EGFR, indicating that EGFR signalling stands not strictly atop the DV patterning genes. Within this network of genetic interactions, Vnd acts as a positive EGFR feedback regulator. Further, we show that EGFR signalling becomes dependent on *single-minded*-expressing midline cells in the posterior brain (tritocerebrum), but remains midline-independent in the anterior brain (deuto- and protocerebrum). Finally, we demonstrate that activated EGFR controls the proper formation of brain neuroblasts by regulating the number, survival and proneural gene expression of neuroectodermal progenitor cells. These data demonstrate that EGFR signalling is crucially important for patterning and early neurogenesis of the brain.

## 1. Introduction

The central nervous system in insects and mammals arises from multipotent neural stem cells that give rise to a vast array of distinct cell types. The underlying molecular genetic mechanisms have been extensively studied in the developing embryonic truncal nervous system (reviewed in [1,2]). In *Drosophila*, the ventral nerve cord (VNC) is generated by segmental arrays of neural stem cells, called neuroblasts, which delaminate from the truncal neuroectoderm (NE). Each neuroblast acquires a unique identity that is reflected by the typical developmental time point and position, and the combination of developmental control genes it expresses [3], which finally determines the number and types of progeny it generates [4,5]. The identity of each neuroblast is initially determined by the combinatorial code of positional cues in the NE, provided by the products of early patterning genes (reviewed in [1]).

A group of genes essentially involved in patterning of the VNC is the evolutionary conserved dorsoventral (DV) genes. Their activity subdivides the NE along the DV axis into longitudinal columns: *ventral nervous system defective* (*vnd*) is expressed in the ventral, *intermediate neuroblasts defective* (*ind*) in the intermediate and *muscle segment homeobox* (*msh*; *Drop* [Dr], FlyBase) in the dorsal neuroectodermal column, where they control the formation of neuroblasts (except *msh*) and specify aspects of their fate [6–16]. The expression domains of DV genes and, accordingly, the DV boundaries of the NE are regulated by the graded activity of the nuclear factor Dorsal and Bone morphogenetic protein [17–19]. Another signalling pathway that controls the regionalized expression of DV genes is the epidermal growth factor receptor (EGFR) pathway [17,20,21], which is highly conserved from fly to human (reviewed in [22,23]). The EGFR pathway is required to induce *ind* expression and to maintain *vnd* expression [17,24,25], and thus to control the formation of intermediate and the identity of ventral neuroblasts [20,24]. Localized EGFR activation depends on the neuregulin-like ligand Vein (Vn) and the TGF-α homologue Spitz (Spi) [26–28]. Spi is processed and secreted by the combined activity of the transmembrane protease Rhomboid (Rho) and the chaperone Star (S), and serves as the cardinal activating ligand [28–31]. Expression of *rho* is tightly controlled and represents the key to the dynamic activation of EGFR, whereas the inactive Spi precursor and S are rather broadly expressed in the NE [32–35]. The spatio-temporal pattern of EGFR activity also depends on the inhibiting ligand Argos (Aos), which is induced in response to high levels of EGFR activity [36], and antagonizes EGFR activation by sequestering Spi [27,37–39]. Although EGFR signalling is initially induced by ligands (Spi, Vn) expressed and secreted from the ventral NE [20,26,27,40], by gastrulation EGFR signalling becomes dependent on Spi, which is secreted from the ventral midline [20,35,41,42].

Similar to the situation in the trunk, it has been shown that EGFR signalling is required in midline cells of the embryonic head, which follow a particular mode of neurogenesis to give rise to the larval visual system, stomatogastric nervous system and most medial parts of the brain [43–46]. However, the regulation of EGFR signalling and its role in neuroectodermal patterning and specification of cell fate along the DV axis are not well understood in the early embryonic brain. The NE of the embryonic brain gives rise to an array of about 100 neuroblasts in each hemisphere, which can be subdivided (from anterior to posterior) into the presumptive proto- (PC), deuto- (DC) and tritocerebrum (TC) [47,48]. Based on a distinct combination of regulatory genes expressed, each brain neuroblast acquires a unique identity [49], which suggests that the expression of patterning genes in the overlying NE has to be precisely controlled during neuroblast formation. In previous reports, we showed that the regionalized expression of DV genes exhibits neuromere-specific differences in the NE and neuroblasts of the embryonic brain [50,51]. We then uncovered a genetic network in which evolutionarily conserved factors encoded by DV genes (*vnd*, *ind*, *msh*, *Nkx6*) and AP patterning genes (*empty spiracles*, *engrailed*) closely interact to properly pattern the NE and specify neuroblast identity along the DV axis of the brain [52,53].

In this study, we shed light on the regulation and function of EGFR signalling in early embryonic brain development. We show that a neuromere-specific deployment of activating (Spi, Vn) and inhibiting (Aos) ligands controls the localized activation of EGFR in the brain NE, which, in turn, is necessary for the spatially restricted expression of all DV genes. We also show that DV genes (*vnd*, *Nkx6*, *ind*) regulate the expression of *vn* and *aos*, which indicates that within the genetic network EGFR stands not strictly atop the DV genes. Moreover, Single-minded (Sim), a master regulator of CNS midline cells [54], is needed for EGFR signalling specifically in TC, but not in the anterior brain (DC, PC). Finally, we show that activated EGFR promotes the formation of brain neuroblasts, as it controls the number, survival and proneural gene expression of neuroectodermal progenitor cells. Thus, EGFR signalling plays a central role in DV patterning and early neurogenesis of the fly brain.

## 2. Results

### 2.1. The pattern of EGFR activity compared with DV gene expression in the early brain

**Figure 1.**
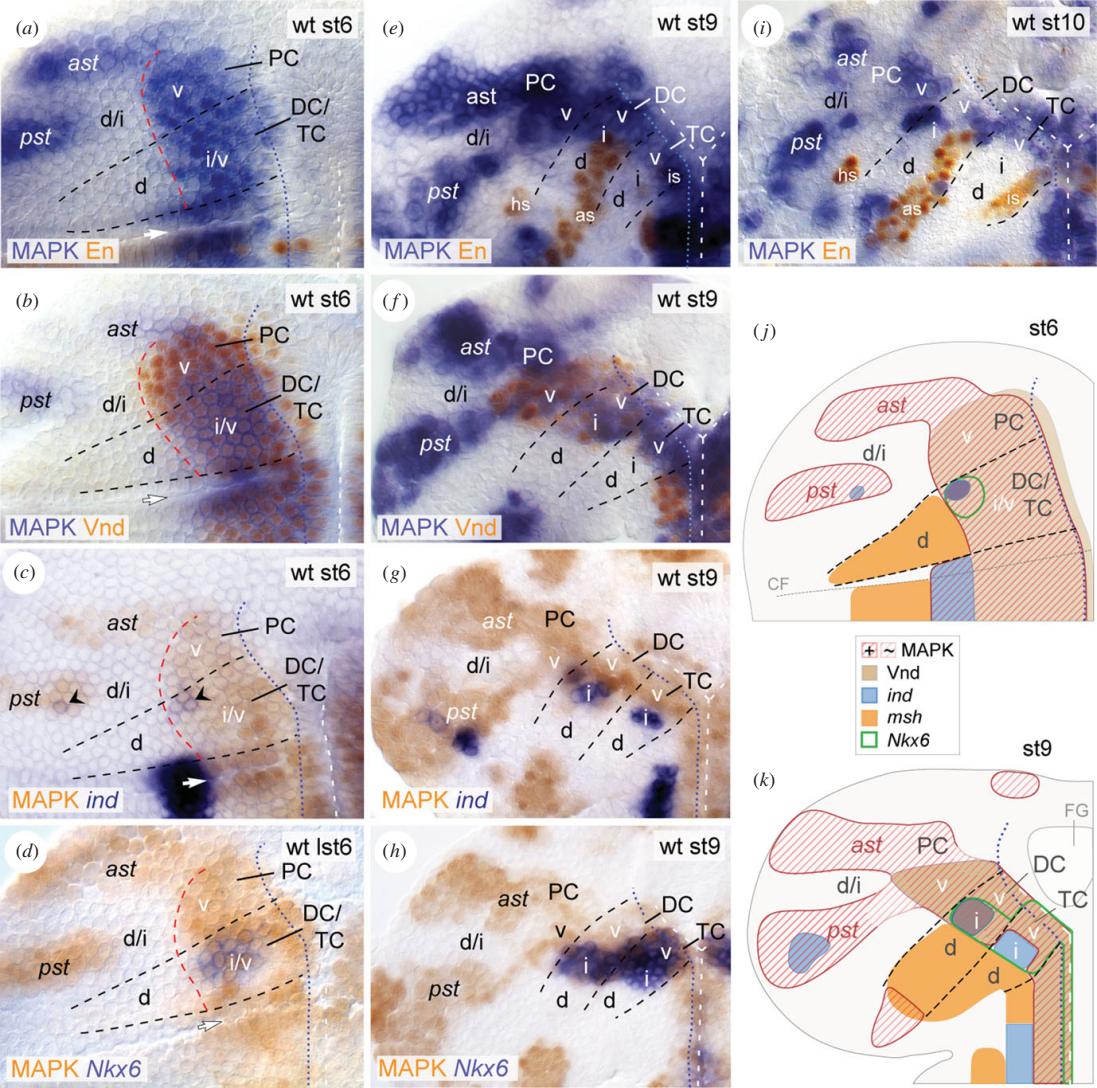
MAPK pattern in the brain NE. (*a–i*) Flat preparations displaying the wild-type (wt) head ectoderm of the left hemisphere at stage 6 (st6) (*a–d*), stage 9 (st9) (*e–h*), and stage 10 (st10) (*i*); anterior is up. MAPK pattern is combined with En (*a,e,i*), Vnd (*b*,*f*), *ind* (*c*,*g*) and *Nkx6* (*d*,*h*). (*c*,*g*) *ind* expression in DC/PC (black arrowheads in (*c*)), but not in TC (*g*) initiates within MAPK-positive NE. (*j,k*) Schematic representation of gene expression patterns in (*a–i*) (also including *msh* expression; see also electronic supplementary material, figure S1). *ast*, anterior and *pst*, posterior protocerebral MAPK stripe. v, ventral; i, intermediate; d, dorsal. Dashed lines in black indicate borders between trito- (TC), deuto- (DC), protocerebrum (PC). At stage 6 (which is slightly prior to the expression of the segmental marker En in the brain NE), tentative boundaries between presumptive brain neuromeres were estimated with regard to the distance from the cephalic furrow (CF; white arrow in (*a–d*)) in AP axis and the AP extent of DV gene expression domains (i.e. *ind* in (*c*) and *Nkx6* in (*d*); see also [52,53]). Dashed lines in white indicate the ventral midline. Dashed lines in red (in (*a–d*)) mark the border between intermediate/dorsal NE in TC/DC, and ventral/intermediate NE in PC. Dotted lines in blue indicate border between NE and mesectoderm. FG, foregut; hs, *en* head spot; as, *en* antennal stripe; is, *en* intercalary stripe. See the main text for further details.

To test whether EGFR controls DV patterning in the early brain, we first compared the activation pattern of EGFR with the known expression patterns of the DV genes *vnd*, *ind*, *Nkx6* and *msh* [52,53]. We visualized EGFR activity in the brain NE by using an antibody against double-phosphorylated (activated) MAPK [27]. Additionally, the segmental marker Engrailed (En) was used to delineate the borders between brain neuromeres (according to [48,50]). MAPK is initiated at stage 5 in a broad longitudinal stripe (data not shown), which by stage 6 precisely overlaps with expression of *vnd*, and includes expression of *ind* and *Nkx6* (figure 1*a–d,j*). Thus, EGFR signalling in the presumptive TC and DC is confined to the ventral and intermediate NE (and ventrally adjacent mesectoderm). In the presumptive PC, MAPK overlaps with Vnd in the ventral NE, but is additionally detected in two large stripes in the intermediate/dorsal NE (the ‘anterior stripe’ and ‘posterior stripe’; according to [45]). By stage 9, MAPK is kept at strong levels in PC, DC and ventral TC, but is largely vanished from the intermediate TC (figure 1*e–h,k*). Interestingly, *ind* expression, which depends on EGFR in the developing VNC [17], is not initiated in the intermediate TC before MAPK has vanished (figure 1*g*), unlike in PC and DC (figure 1*c*). From early stage 10 onwards, MAPK becomes confined to smaller cell clusters in the brain NE (figure 1*i*). During stages 5–11, MAPK remains complementarily expressed to *msh* (figure 1*j,k*; electronic supplementary material, figure S1*a,b*). EGFR is therefore not active in the dorsal NE of TC and DC. During stages 8–11, MAPK is also transiently detected in subsets of neuroblasts that mostly develop from the MAPK-positive brain NE (electronic supplementary material, figure S2*a,b*).

### 2.2. EGFR is required for neuromere-specific expression of *ind*, *vnd* and *Nkx6*

**Figure 2.**
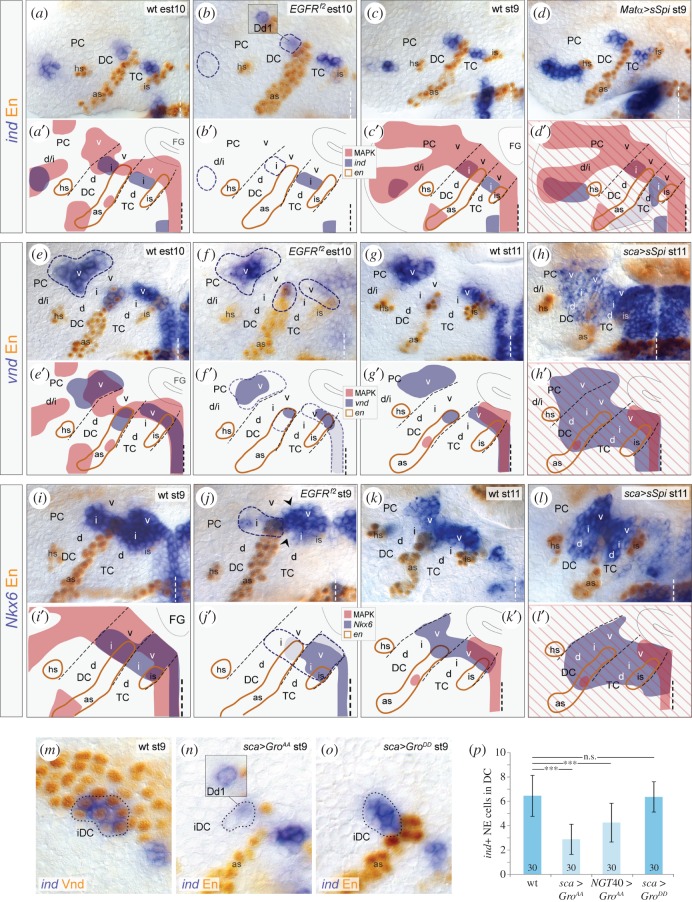
EGFR controls expression of *ind*, *vnd* and *Nkx6*. (*a–l*) Double stainings against *ind* (*a–d*), *vnd* (*e–h*), or *Nkx6* mRNA (*i–l*) and Engrailed protein in different genetic backgrounds. Panels (*a′–l′*) show schematics of (*a–l*). (*a,a′,b,b′*) *ind* expression in the NE of DC/PC, but not TC, is strongly reduced in *EGFR^f2^* mutants at early stage 10 (est10) (hatched areas in (*b*) indicate NE with a loss of *ind* expression), compared with wild-type (*a*). Note, *ind* expression is not affected in deutocerebral neuroblast Dd1 (inset in (*b,b′*)). (*c,c′,d,d′*) At stage 9, *ind* expression is dorsally expanded upon *Matα* *>* *sSpi* in TC/PC, but not in DC. (*e,e′,f,f′*) *vnd* expression is reduced in all neuromeres of *EGFR^f2^* mutants at early stage 10 (areas are encircled with hatched line in (*f,f′*). (*g,g′,h,h′*) At stage 11, *vnd* expression is ectopically detected in the entire DC and intermediate/dorsal NE of TC upon *sca* *>* *sSpi*. (*i,j′*) At stage 9, *Nkx6* expression is reduced in the *EGFR^f2-^*mutant intermediate DC (area encircled with a dashed line in (*h*)) and in a few cells in the intermediate/ventral TC (arrowheads in (*h*)). (*k,k′,l,l′*) *Nkx6* expression is expanded into the intermediate and dorsal NE of TC/DC upon *sca* *>* *sSpi* at stage 11. (*m*) *ind* and Vnd are coexpressed in intermediate DC (encircled) at stage 9. (*n*) In *sca* *>* *Gro^AA^* background, *ind* expression is reduced in the deutocerebral neuroectoderm, while unaffected in Dd1 (inset). (*o*) *ind* is not reduced upon *sca* *>* *Gro^DD^*. (*p*) At stage 9, *ind*-expressing neuroectodermal cells in DC are significantly reduced after *sca* *>* *Gro^AA^* (2,8 ± 1,2 cells) and *NGT40* *>* *Gro^AA^* (4,3 ± 1,6 cells), but not after *sca* *>* *Gro^DD^* (6,4 ± 1,3 cells), compared with wild-type (wt) (6,5 ± 1,7 cells) (*n* = 30 each); error bars indicate s.d.; ***p < 0.0001; n.s., not significant; unpaired Student's *t*-test. For orientation and other abbreviations see figure 1.

Because EGFR activity overlaps with the expression domains of *vnd*, *Nkx6 and ind* in the brain NE*,* we tested whether EGFR controls the expression of these genes. Analysing *EGFR^f2^* mutants, we found that *ind* expression is delayed and strongly reduced in DC and PC (87% and 100% of brain hemispheres, respectively; *n* = 35; figure 2*a,b*), or entirely missing in DC (13% of hemispheres; *n* = 35). *ind* levels in TC of *EGFR^f2^* mutants seemed to be unaffected, being consistent with *ind* expression complementary to MAPK in wild-type embryos. Ectopic activation of EGFR by overexpression of the secreted (i.e. active) EGFR ligand Spitz (*sSpi*) using the maternal *Matα*-Gal4 line (which drives expression ubiquitously; in the following termed *Matα* *>* *sSpi*) [55] led to ectopic activation of *ind* in intermediate/dorsal PC and dorsal TC (100% and 25% of hemispheres, respectively; *n* = 32), while *ind* was not affected in DC (figure 2*c,d*). Thus, EGFR signal is necessary for *ind* expression in DC and PC, and sufficient to activate *ind* expression in TC and PC.

Expression of *vnd* expression was activated normally in *EGFR^f2^* mutants, but strongly reduced at early stage 10 in the NE of TC, DC (100% and 83% of hemispheres, respectively; *n* = 25) and, to a lower extent, PC (83% of hemispheres; *n* = 25; figure 2*e,f*). To ectopically activate EGFR, we misexpressed sSpi using the *scabrous (sca)-*Gal4 line [56] which drives expression within the NE stronger than the maternal driver used in this study. Because *sca*-Gal4 induces misexpression not before stage 7, thus later than the maternal driver, we usually took this driver to investigate effects at later stages (stages 11–13). In these *sca* *>* *sSpi* embryos, *vnd* was ectopically expressed in TC and DC, but not in PC (88% and 79% of hemispheres, respectively; *n* = 48; figure 2*g,h*). We conclude that EGFR is necessary for the maintenance and sufficient for induction of *vnd* expression especially in TC and DC. As with *vnd*, we observed similar effects of EGFR on the expression of *Nkx6* in TC and DC (figure 2*i–l*). However, we did not observe effects of EGFR on the expression of *scarecrow,* another *Nkx* gene with close homology to *vnd* [57] (data not shown).

In TC and DC, we furthermore observed a slight derepression of *msh* in *EGFR^f2^* mutants, and conversely, a reduction of *msh* expression in *Matα* *>* *sSpi* embryos (electronic supplementary material, figure S3*a–d*). To test whether EGFR signal directly represses *msh*, we analysed patterns of *msh* and MAPK (which normally exclude each other) in *vnd^6^* mutants; in these mutants, *msh* was derepressed in the intermediate/ventral NE, and MAPK remained unaffected, both factors now being largely coexpressed in this NE (electronic supplementary material, figure S3*e,f*). These results suggest that activated EGFR regulates *msh* indirectly, through the activity of other DV genes *vnd*, *ind* and *Nkx6* (as shown above).

### 2.3. EGFR induces *ind* expression in DC by phosphorylation of the co-repressor Groucho

*vnd* and *ind* are exceptionally coexpressed in the intermediate DC [51] (figure 2*m*), where EGFR is also activated. EGFR can regulate gene expression by MAPK-dependent phosphorylation and thus inactivation of the co-repressor Groucho (Gro) [58–60]. As Vnd is a Gro-dependent repressor [61], we hypothesized that EGFR signal phosphorylates Gro and thus inactivates Vnd/Gro-mediated repression of *ind*. To test this, we analysed *ind* expression after ectopically expressing unphosphorylatable (i.e. constitutively active; Gro^AA^) and pseudo-phosphorylated (inactive; Gro^DD^) Gro-constructs [58] in the brain NE using the *sca*-Gal4 and maternal *NGT40*-Gal4 [62] driver lines, which both drive Gal4 expression in the NE (although *NGT40*-Gal4 induces misexpression earlier and at weaker levels than *sca*-Gal4; data not shown). *ind* was significantly reduced in DC of *sca* *>* *Gro^AA^* and *NGT40* *>* *Gro^AA^* embryos (figure 2*n,p*; data not shown), with effects being stronger with the later-initiating but stronger driver *sca-Gal4*, but unaltered in DC of *sca* *>* *Gro^DD^* control embryos (figure 2*o,p*). Altogether, our results strongly suggest, that EGFR activity induces *ind* expression in DC specifically by phosphorylation of Gro, thus inactivating Vnd/Gro.

### 2.4. *rho, vn* and *S* are differentially expressed in the brain neuroectoderm and flanking mesectoderm

**Figure 3.**
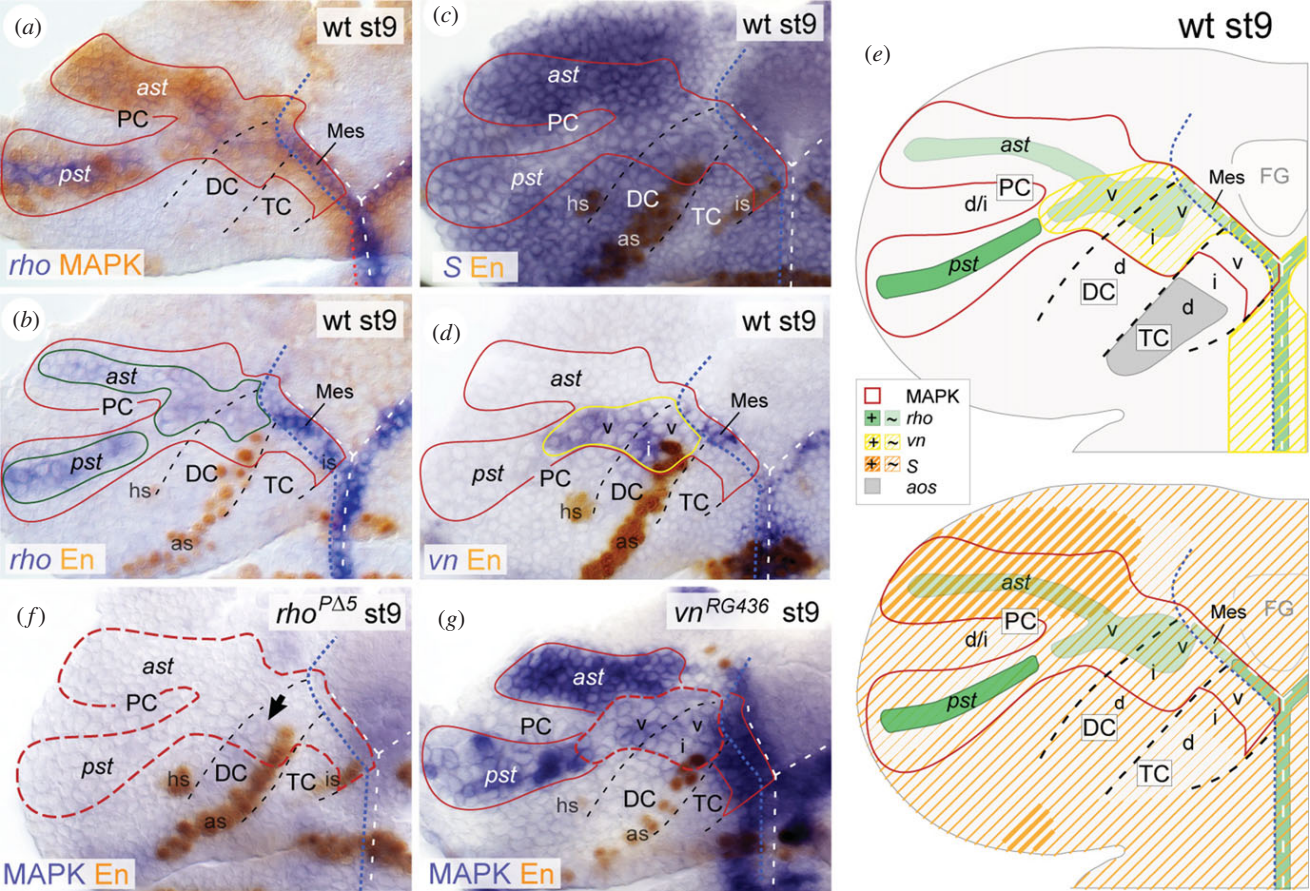
*rho*, *vn*, *S* and *aos* are differentially expressed and required in distinct regions of the brain. Stage 9 (st9); red solid line demarcates the wild-typic MAPK domain, according to (*a*). (*a*,*b*) *rho* is expressed within MAPK domain in DC/PC (solid green line), and in the mesectoderm (‘midline cells’) ventral to TC/DC which are characterized by expression of Single-minded (shown in figure 5*n*,*q*). (*c*) *S* is broadly expressed in the brain NE, while expression levels vary. (*d*) *vn* is expressed in ventral PC and ventral/intermediate DC (solid yellow line), and mesectodermal cells. (*e*) Scheme summarizes expression data in (*a–d*), and the EGFR-independent expression of *aos* in dorsal TC (see also figure 4*g* and the main text for further explanations). (*f*) MAPK is strongly reduced in *rho^PΔ5^* mutant brain NE (red hatched line), when compared with wild-type (*a*); black arrow indicates faint MAPK in the mutant DC. (*g*) In *vn^RG436^* mutants, MAPK is reduced particularly in the ventral PC, and ventral/intermediate DC (red hatched line). For orientation, other abbreviations and symbols see figure 1.

The complex spatio-temporal pattern of EGFR activation in the brain NE led us to determine the sources of activating Spi and Vn ligands. Spi is expressed as an inactive precursor and requires Rho and S to be secreted [28–31]. *spi* itself and *S* are rather broadly expressed, thus playing a minor role in controlling the spatio-temporal pattern of Spi secretion [32–35]. By contrast, *rho* expression is tightly controlled and represents the key regulatory step controlling Spi secretion in the developing VNC [35,40]. As we cannot exclude that the spatio-temporal Spi secretion is also regulated by *spi* and *S* expression in the early brain, we analysed *rho*, *spi* and *S* expression to determine Spi ligand sources in the brain NE. At stage 5, *rho* expression in the NE corresponded with the MAPK pattern, but became largely restricted to the *sim*-expressing mesectoderm by gastrulation (at stage 6), although rudimentary *rho* expression was still detectable in the NE of DC and PC (electronic supplementary material, figure S4*a,b*). By stage 9, when EGFR signalling and DV regionalization of the brain NE are most pronounced (figure 1) [52], *rho* expression was weakly detected in DC and ventral PC, and the anterior MAPK stripe of PC; stronger *rho* expression was observed in the posterior MAPK stripe and mesectoderm (ventral to TC and DC) (figure 3*a,b*). Thus, *rho* expression closely correlates with EGFR activity, even though *rho* expression levels vary within the brain NE and flanking mesectoderm. *S* and *spi* were broadly expressed in the brain NE (figure 3*c*; and data not shown). We noted that *S* expression was particularly strong in the anterior MAPK stripe of PC, where *rho* expression was weak, despite strong EGFR signalling (figure 3*a–c*). In contrast with Spi, Vn is expressed as a secreted protein which requires no further processing to be activated [26]. *vn* expression was codetected with MAPK in DC, ventral PC and mesectoderm (ventral to TC and DC) at stage 9 (figure 3*d*; electronic supplementary material, figure S4*c*). Because *vn* expression was never observed in both MAPK stripes of PC, this suggests that Vn only partially acts as a positive EGFR feedback regulator in the brain, in contrast with the VNC [42].

In sum, *rho*, *vn* and *S* are differentially expressed during patterning of the brain (summarized in figure 3*e*). Primarily *vn* and little *rho* is expressed in DC and abutting ventral PC. By contrast, *rho* and *S* are expressed in the two protocerebral MAPK stripes, with weak *rho* and strong *S* expression levels in the anterior stripe, opposite to the posterior stripe. Only *S*, but not *vn* or *rho*, is expressed in the TC, although the adjacent mesectoderm reveals strong *vn*/*rho* expression levels. These data suggest that EGFR activity in the brain is regulated by a region-specific deployment of distinct ligands.

### 2.5. *rho* and *vn* are differentially required to activate EGFR in the brain

To test for requirements of Rho and Vn to activate EGFR, we analysed EGFR activity in the brain NE of *rho^PΔ5^* and *vn^RG436^* mutant embryos. At stage 9, *vn^RG436^* mutants exhibited a specific loss of MAPK only in DC and ventral PC (strong reduction in 40%, moderate reduction in 49%, normal in 11% of hemispheres; *n* = 35), whereas *rho^PΔ5^* mutants showed a near total loss of MAPK in the entire brain NE (100% of hemispheres; *n* = 22) (figure 3*f,g*). We conclude that Rho is required for EGFR activation in the entire brain NE, whereas Vn is required in addition to Rho for proper EGFR activation in DC and ventral PC. As MAPK was lost in DC/ventral PC in both, *rho* and *vn* mutants, we asked if *vn* expression depends on Rho. Indeed, *vn* expression was reduced in these brain regions in *rho^PΔ5^* mutants (electronic supplementary material, figure S4*d*), indicating that Rho-dependent factors normally induce *vn* expression, which in turn activates EGFR. However, we cannot rule out that low levels of Rho act in parallel to Vn to achieve proper EGFR activation in DC and ventral PC.

### 2.6. *vn* expression is controlled by Vnd in the brain

**Figure 4.**
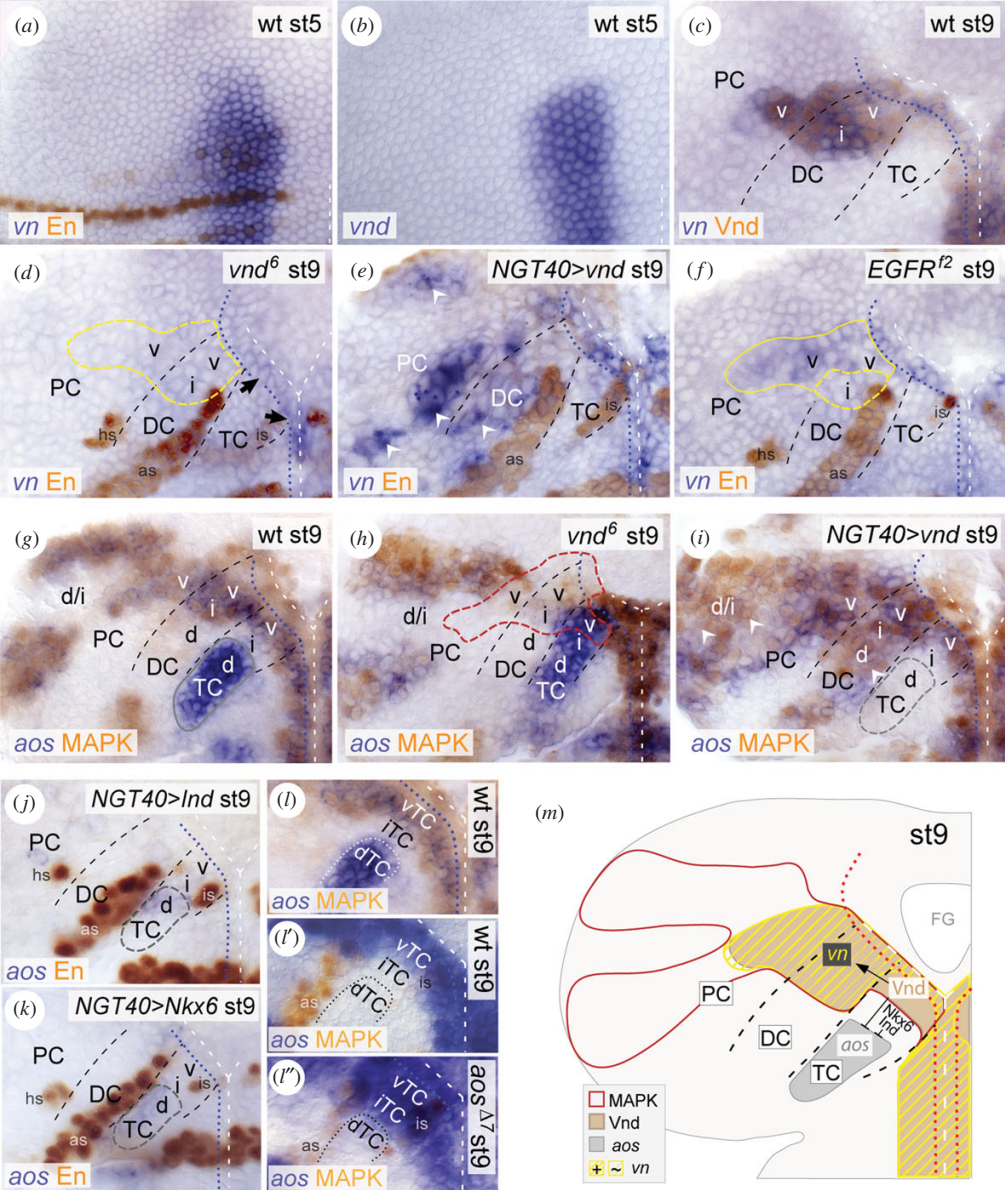
Vnd controls EGFR activity by regulating expression of *vn* and *aos*. (*a*,*b*) Stage (st) 5, (*c–l*) stage 9. (*a–c*) In the presumptive brain NE, *vn* expression closely corresponds to *vnd* expression between stages (st) 5 to 9. (*d–f*) *vn* expression is lost in the brain NE in *vnd^6^* mutants (yellow dashed outline in (*d*)); only a few mesectodermal cells ventral to TC remain *vn*-positive (black arrows in *d*). (*e*) Upon *NGT40* *>* *vnd*, *vn* is ectopically induced in dorsal DC and PC (white arrowheads). (*f*) *vn* expression is reduced in the brain NE of *EGFR^f2^* mutants (predominantly in intermediate DC; yellow dashed outline). (*g–i*) In wild-type, *aos* is strongly expressed in the MAPK-negative dorsal TC (and weaker within the MAPK domains; see below) (*g*). (*h*) In *vnd^6^* mutants, *aos* is de-repressed in ventral/intermediate TC, and MAPK reduced in ventral TC and in the adjacent NE of DC and PC (red dashed outline). (*i*) Upon *NGT40* *>* *vnd*, *aos* is specifically missing in dorsal TC (grey dashed outline), while MAPK is ectopically detected in dorsal DC and PC (white arrowheads). Note that in these different genetic backgrounds at stage 9 *aos* is always co-detected with MAPK in the brain NE, because *aos* is induced downstream of EGFR/MAPK [36], except in dorsal TC (see also electronic supplementary material, figure S4*j,k*). Accordingly, upon *NGT40* *>* *vnd* ectopic *aos* expression within the enlarged MAPK domain (in (*i*)) is most probably due to ectopic EGFR activation. (*j,k*) *aos* is strongly reduced in dorsal TC (grey dashed outline) upon *NGT40* *>* *ind* (J) or *NGT40* *>* *Nkx6* (*k*). (*l*) EGFR is inactive in intermediate TC, which adjoins *aos* expression in dorsal TC (dotted outline in (*l–l″*)). In *aos^Δ7^* mutants, EGFR is ectopically activated in intermediate TC. (*m*) Schematic summary. Vnd activates *vn* expression in the ventral brain NE, and represses *aos* in ventral TC, whereas Ind/Nkx6 repress *aos* in intermediate TC. For orientation, other abbreviations and symbols see figure 1.

We found that expression of *vn* closely correlates with expression of *vnd* in the early brain NE (figure 4*a–c*). Therefore, we tested if Vnd controls *vn* expression. At stage 9, *vnd^6^*-mutant embryos showed strong reduction of *vn* expression in the brain NE (figure 4*d*). Correspondingly, we found ectopic *vn* expression in DC and PC upon *vnd-*overexpression (*NGT40* *>* *vnd*) (figure 4*e*). These data suggest that Vnd is necessary, and to some extent sufficient, to activate *vn* expression. Next, we tested if *vn* expression is induced by activated EGFR, as it has been proposed in the VNC [42]. At stage 9, we observed a slight reduction of *vn* expression in the brain NE of *EGFR^f2^* mutants (figure 4*f*), compared with the strong reduction in *vnd^6^* mutants. Further, in *EGFR^f2^* mutants, the reduction of *vn* expression was closely correlated with the reduction of *vnd* expression (as shown above); residual *vn* expression was always co-detected with residual *vnd* expression (electronic supplementary material, figure S4*e*). This suggests that in the brain, EGFR induces *vn* expression indirectly, via Vnd. Our data thus provide the first evidence that Vnd, a DV gene which has been considered as an EGFR target so far, regulates the expression of an EGFR ligand.

### 2.7. *aos* is expressed in dorsal TC independently of EGFR but under control of Vnd, Ind and Nkx6, and inhibits EGFR signalling

Argos (Aos) is a secreted EGFR regulator that antagonizes Spi activity [38]. We detected *aos* expression within the neuroectodermal MAPK domains in all brain neuromeres (figure 4*g*), which was expected because *aos* is known as a negative EGFR feedback regulator induced downstream of EGFR/MAPK [36]. Consistently, *aos^Δ7^* mutants showed a massive overactivation of MAPK in the brain NE at stage 11, when endogenous EGFR activity is largely downregulated (electronic supplementary material, figure S4*f,g*). Interestingly, we recognized a prominent *aos* expression domain outside the MAPK pattern, in dorsal TC (figure 4*g*; electronic supplementary material, figure S4 *h,i*). Accordingly, this *aos* domain was not affected in *EGFR^f2^* embryos, whereas the remaining *aos* expression was entirely missing in the brain (electronic supplementary material, figure S4*j,k*). We conclude that *aos* is regulated independently of EGFR in dorsal TC.

Expression of *aos* restricted to dorsal TC suggests a regulation by DV genes. Therefore, we tested whether *vnd*, *ind* or *Nkx6,* expressed in ventral/intermediate TC (see §2.1.), act to restrict *aos* to the dorsal TC. In *vnd^6^* mutants, which are characterized by an additional loss of *ind* and *Nkx6* in the TC [52] (electronic supplementary material, figure S4*l,m*), *aos* is derepressed in the ventral/intermediate TC (figure 4*h*). Conversely, *aos* is efficiently repressed in *NGT40* > *vnd* embryos (lost in 87%, strongly reduced in 13% of hemispheres; *n* = 30) (figure 4*i*). We did not find substantial effects on *aos* expression in *ind* or *Nkx6* mutants (data not shown), most probably because both factors act redundantly [52]. However, overexpression of either *ind* or *Nkx6* with *NGT40*-Gal4 led to a strong reduction of *aos* expression in dorsal TC (figure 4*j,k*). We conclude that *vnd*, *ind* and *Nkx6* act in concert to restrict *aos* to dorsal TC. This provides further evidence of DV patterning genes regulating the regionalized production of EGFR ligands.

Being controlled by the DV gene network, we asked whether Aos is involved in regulating EGFR activity along the DV axis in TC. At stage 9, EGFR is activated only in ventral TC. However, in *aos^Δ7^* mutants, EGFR was additionally activated in intermediate TC (figure 4*l–l″*), indicating that Aos normally inhibits EGFR signalling in this NE. Thus, Aos is crucial for regulation of EGFR activity along the DV axis in TC.

### 2.8. Vnd is part of a positive feedback loop controlling EGFR activity in the brain

As Vnd controls the activity of both activating EGFR ligands, Vn and Spi (via *aos*, as shown above), we analysed the effect of Vnd on EGFR activity in the brain NE. At stage 9, *vnd^6^* mutants exhibited a reduction of MAPK in TC, DC and adjacent ventral PC (figure 4*h*). In TC, this reduction is probably due to the ventrally expanded domain of *aos* expression which inhibits Spi-induced EGFR activation. In the *vnd^6^*-mutant DC/ventral PC, elevated Aos levels, secreted from the enlarged *aos* domain in TC, come together with the loss of Vn (figure 4*d,h*), which we propose to mutually account for the strong reduction of EGFR activation. To test whether Vnd is sufficient to induce EGFR, we analysed MAPK after *vnd*-overexpression. In *NGT40* > *vnd* embryos, we found ectopic MAPK in PC and dorsal DC (figure 4*i*). We assume that EGFR is overactivated in PC primarily due to ectopic Vn (figure 4*e*), and in DC due to elevated levels of Vn and Spi, the latter owing to the loss of Aos in dorsal TC (figure 4*e,i*).

Taken together, these data demonstrate that Vnd plays a central role for the spatial activity of EGFR as it controls the ligand activity of Vn and Spi (figure 4*m*). As the maintenance of *vnd* expression in turn depends on activated EGFR (as shown in §2.2.), Vnd acts as a positive EGFR feedback regulator in the brain.

### 2.9. EGFR signalling crucially depends on Tll in PC, and on mesectodermal Sim in TC

**Figure 5.**
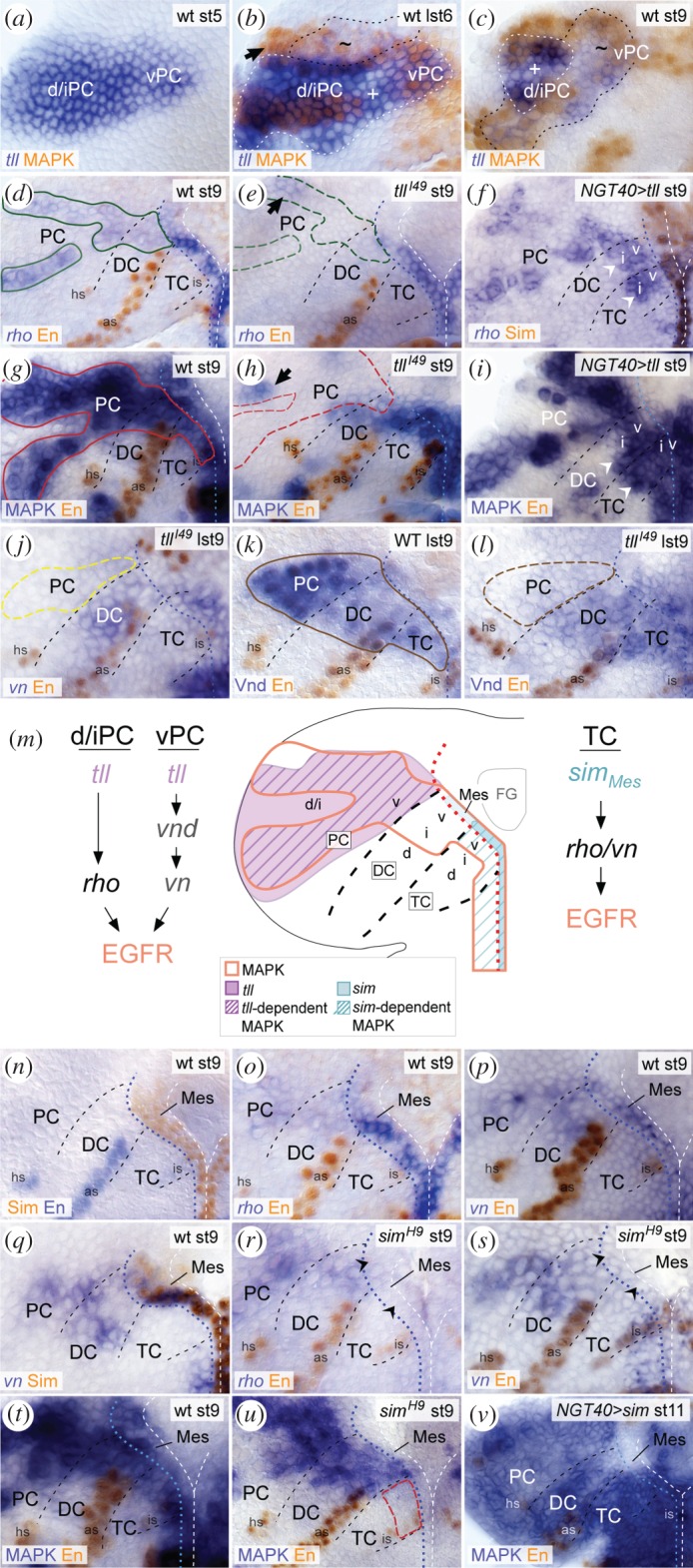
EGFR activity depends on Tll in PC, and on Sim in TC. (*a*–*c*) At stage 5 (st5) *tll* expression is activated exclusively in PC [63], prior to MAPK (*a,b*). (*b*) At late stage 6 (lst6), the protocerebral MAPK domain largely originates within the *tll* domain (encircled with black dashed line); indicated are subregions of strong (+) and weak (∼) *tll* expression levels. Few MAPK-positive cells (arrow) originate outside the *tll*-expressing NE. (*c*) Until stage 9, *tll* expression largely overlaps with MAPK in PC. (*d*–*f*) At stage 9, *rho* expression is missing in *tll^l49^* mutant PC (green dashed outline in (*e*)), except in anteriodorsal NE (arrow), when compared with wild-type (solid green outline in (*d*)). (*f*) Upon *NGT40* *>* *tll, rho* is ectopically expressed in ventral TC/DC (white arrowheads), and detected at stronger expression levels in ventral PC. Note that En expression is repressed in *NGT40* *>* *tll.* Tentative segment boundaries were therefore estimated based on their wild-typic position with regard to the cephalic furrow, because the extent of the brain NE is largely unaffected in *NGT40* *>* *tll* embryos. (*g–i*) At stage 9, compared to wild-type (*g*), MAPK is missing in *tll^l49^* mutant PC (red hatched outline) in 80% of hemispheres (*n* = 20) (*h*) and ectopically activated in ventral/intermediate TC and DC upon *NGT40* *>* *tll* (white arrowheads in (*i*)). We cannot exclude that lack of *en* expression in TC and DC partly evokes ectopic MAPK or *rho* expression (*f*) in these neuromeres. (*j–l*) At late stage 9, *vn* expression is lost in PC (yellow hatched outline; compared with wild-type in (*p*)). (*l*) Correspondingly, Vnd is lost in PC of *tll^l49^* mutants (brown hatched outline), compared with wild-type (brown solid line in (*k*)). (*m*) Schematic summary of data in (*a–l*) and (*n–v*). Neuroectodermal Tll (violet) induces EGFR signalling in PC (hatched in violet), while mesectodermal Sim (turquoise) induces EGFR activity in ventral TC (hatched in turquoise). (*n–s*) At stage 9, coexpression of Sim (*n,q*), *rho* (*o*) and *vn* (*p,q*) in mesectodermal cells ventral to TC/DC. Expression of *rho* (*o*) and *vn* (*p,q*) is lost in *sim^H9^* mutant mesectoderm (black arrowheads in *r,s*). (*b*) MAPK is specifically lost in TC (red hatched line) and mesectoderm of *sim^H9^* mutants (*u*), and upon *NGT40* *>* *sim* ubiquitously activated in the brain NE at stage 11 (*v*). For orientation, other abbreviations and symbols see figure 1.

As EGFR activity in PC emerges largely in the *tailless* (*tll*) expression domain (figure 5*a–c*), we analysed if Tll regulates EGFR activity. *tll^l49^* mutants exhibited a loss of *rho* in the intermediate/dorsal PC while *tll*-overexpression (*NGT40* *>* *tll)* induced ectopic *rho* expression predominantly in ventral/intermediate TC and DC, but not in PC where endogenous Tll is expressed (figure 5*d–f*). Consistent with these results, we detected in corresponding brain regions a loss of MAPK in *tll^l49^* mutants and ectopic MAPK in *NGT40* *>* *tll* embryos (figure 5*g–i*; electronic supplementary material, figure S4*n*). Further, we observed a loss of *vn* expression in ventral PC of *tll^l49^* mutants (figure 5*j*). As Vnd, necessary for *vn* expression (as shown in §2.6.), is also lost in these mutants (figure 5*k,l*), we conclude that in ventral PC, Tll normally induces *vn* expression via Vnd. Taken together, in PC, Tll induces production of both ligands, Vn (in ventral PC) and Spi (in intermediate/dorsal PC), by regulating the expression of *vnd* and *rho*, respectively (figure 5*m*).

Next, we analysed the role of single-minded (Sim), the main regulator of midline-dependent Spi secretion in VNC patterning [35,64,65], in controlling EGFR activity in the early brain. Onset of EGFR signalling occurs independently of Sim, because expression of *rho* initiates in the brain NE (at stage 5) before *sim* in the ventral mesectoderm (at stage 6) (electronic supplementary material, figure S4*a,b*). By stage 9, *sim* is expressed in the mesectoderm ventral to TC and DC (figure 5*n*), where it overlaps with *rho* and *vn* expression (figure 5*o,p,q*). Analysis of *sim^H9^* mutants revealed a loss of *rho*, *vn* and MAPK signal in this mesectoderm (figure 5*r–u*), accompanied by a loss of MAPK specifically in TC (figure 5*t,u*). This indicates that Sim is necessary for the production of Spi and Vn secreted from the mesectoderm to induce EGFR signalling in ventral TC, but is dispensable in DC and PC. Furthermore, *sim* overexpression (*NGT40* *>* *sim*) was sufficient to induce ectopic MAPK in the brain NE by stage 11 (figure 5*v*), probably due to action of *rho* and *vn*, which both can be ectopically induced by Sim in the trunk [65]. Altogether these data suggest that mesectodermal Sim is normally important for ongoing EGFR signalling exclusively in TC.

### 2.10. EGFR controls formation of brain neuroblasts by regulating number, survival and proneural gene expression of neuroectodermal progenitor cells

**Figure 6.**
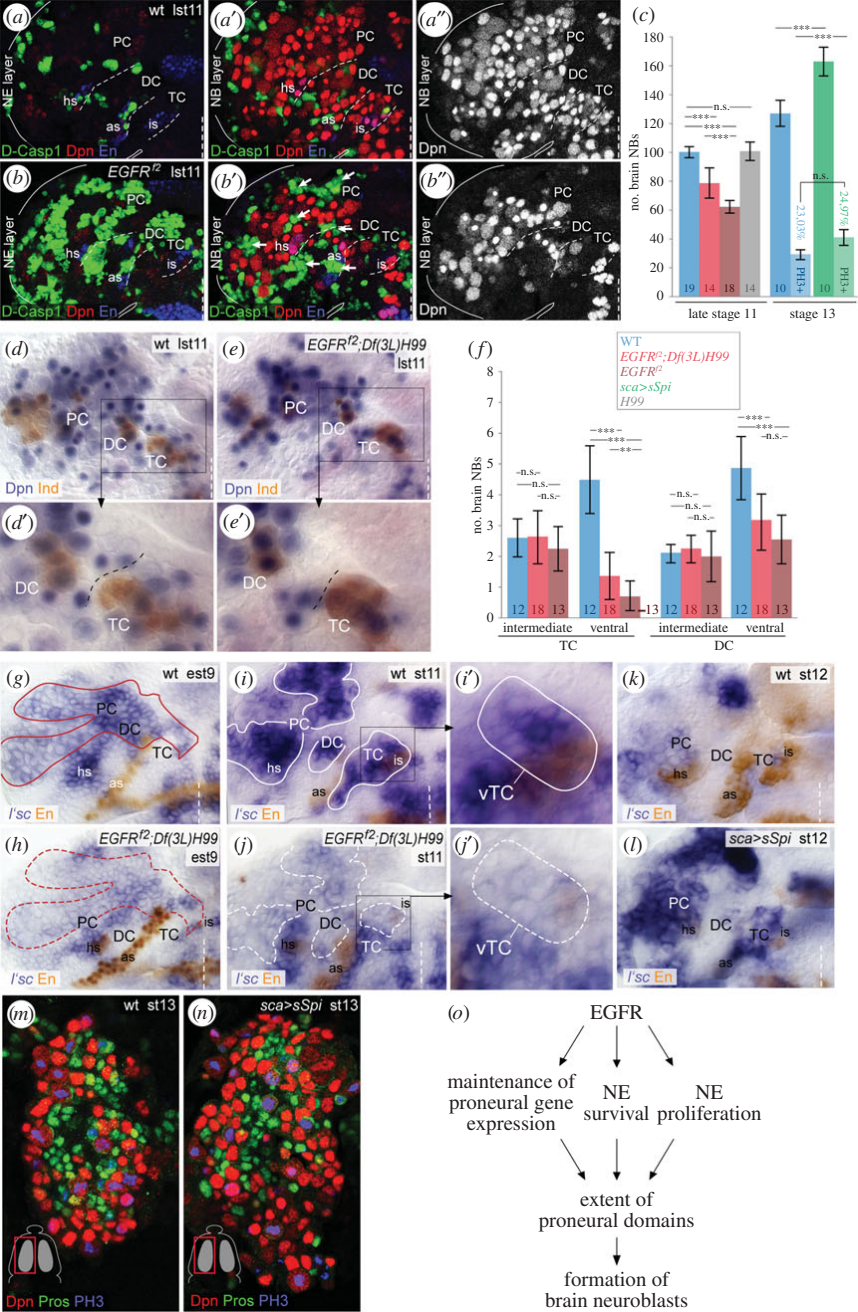
EGFR signalling is crucial for brain neuroblast formation. (*a,a′,a″,b,b′,b″*) Flat preparations of wild-type (*a,a′,a″*) and *EGFR^f2^* mutants (*b,b′,b″*) at late stage 11 (lst11). The wild-typic and mutant NE (*a,b*) or neuroblast (NB) (*a′*,*a″*,*b′*,*b″*) layer depicted in these panels each represent a combined projection of an equal amount of confocal foci (in *Z*-axis). Dcp1-signal is broadly detected in the brain NE (*a,b*) of *EGFR^f2^* mutants, but not in underlying neuroblasts (*a′,a″,b′,b″*). The brain neuroblast number is strongly reduced in the *EGFR^f2^*-mutant hemisphere, in which totally 56 neuroblasts were counted (in *b′*,*b″*), when compared with 104 neuroblasts in the wild-type hemisphere (in *a′*,*a″*). Note that at late stage 11 almost the entire NE undergoes cell death in *EGFR^f2^* mutants. As the neuroectodermal layer dissolves, apoptotic neuroectodermal cells come to lie within the neuroblast layer (white arrows). (*c*) Quantification of the number of brain neuroblasts in different genotypes. Number of neuroblasts per hemisphere at stage 11: wt 100.3 ± 4.1; *EGFR^f2^* 62.3 ± 4.7; *EGFR^f2^;Df(3L)H99* 78.8 ± 10.8; *H99* 100.8 ± 6.7; at stage 13: wt 127.2 ± 9.2 (of those 29.0 ± 3.5 neuroblasts [23.0%] are PH3-labelled); *sca* *>* *spi* 163.4 ± 11.0 (of those 40.8 ± 5.5 neuroblasts [25.0%] are PH3-labelled); numbers within bars indicate *n*; error bars indicate s.d.; ***p < 0.0001, n.s., not significant; unpaired Student's *t*-test). (*d,e*) At late stage 11, the number of Dpn-positive brain neuroblast is significantly reduced in *EGFR^f2^;Df(3L)H99* mutants. (*d′,e′*) Higher magnification of areas boxed in (*d,e*). The number of ventral (black asterisks) but not of (Ind-positive) intermediate neuroblasts (white asterisks) is reduced in TC/DC of those mutants. (*f*) Quantification of the number of intermediate/ventral neuroblasts in TC/DC (at stage 11) in different genotypes. Number of neuroblasts/hemisphere at stage 11: intermediate neuroblasts in TC: wt 2.6 ± 0.6; *EGFR^f2^;H99* 2.6 ± 0.9; *EGFR^f2^* 2.2 ± 0.7; intermediate neuroblasts in DC: wt 2.1 ± 0.3; *EGFR^f2^;H99* 2.3 ± 0.5; *EGFR^f2^* 2.0 ± 0.8; ventral neuroblasts in TC: wt 4.5 ± 1.1; *EGFR^f2^;H99* 1.4 ± 0.8; *EGFR^f2^* 0.7 ± 0.5; ventral neuroblasts in DC: wt 4.9 ± 1.0; *EGFR^f2^;H99* 3.2 ± 0.9; *EGFR^f2^* 2.6 ± 0.8); numbers within bars indicate *n*; error bars indicate s.d.; **p < 0.01, ***p < 0.0001, n.s., not significant; unpaired Student's *t*-test). (*g*,*h*) Wild-typic MAPK domain is outlined in red in (*g)*, and for comparison in the *EGFR^f2^;H99* mutant *(h*). *l'sc* expression is reduced in PC, DC and ventral TC particularly in the *EGFR^f2^*-mutant domain (indicated in (*h*)) at early stage 9 (est9). (*i,i′,j,j′*) Loss of L'sc expression at stage 11 (white hatched outlines in (*i*)), when compared with wild-type (white solid outline in (*j*)). (*i′,j′*) Higher magnification of NE in ventral TC discloses loss of *l'sc* expression, corresponding to the loss of ventral neuroblasts (see (*e′*)). (*k,l*) *l'sc* expression is widely downregulated after formation of brain neuroblast at stage 12 in wild-type (*k*), but maintained upon *sca* *>* *sSpi* (*l*). (*m,n*) Dorsal view on left hemisphere. Number of Dpn-positive/Pros-negative (Pros indicates ganglion mother cells) brain neuroblasts is increased by stage 13 in *sca* *>* *sSpi* embryos (*n*), when compared with wild-type (*m*); the mitotic index of neuroblasts (as judged by PH3-labelling) is unaltered (see (*c*)). (*o*) EGFR controls formation of brain neuroblast in multiple ways (see the main text). For orientation, other abbreviations and symbols see figure 1.

Having established the regulation of EGFR signalling and its interaction with DV patterning genes in the procephalic NE, we asked if EGFR signal functions in the formation of brain neuroblasts. In stainings against Death caspase-1 (Dcp-1), a hallmark of cell death, we recognized extensive cell death in the brain NE (figure 6*a,b*; electronic supplementary material, figure S5*a–d*), which is accompanied by a significant loss of Deadpan (Dpn)-positive brain neuroblasts in all three neuromeres of *EGFR^f2-^*mutant brains at stage 11, when compared with wild-type (figure 6*a′,a″,b′,b″,c*). However, we did not detect apoptotic brain neuroblasts, although EGFR is transiently active in a subset of them (electronic supplementary material, figure S2*a,b*). To test if the loss of neuroblasts is caused by cell death of NE progenitor cells, we estimated the neuroblast number in *EGFR^f2^;Df(3L)H99* double mutants which are cell death deficient [66]. In total, 20–25% of brain neuroblasts were lost in these double mutants, instead of 40% in *EGFR^f2^* mutants (figure 6*c–e′*; electronic supplementary material, figure S5*e,f*), whereas neuroblast numbers were unaffected in *Df(3L)H99* mutant control brains (electronic supplementary material, figure S5*g*,*h*). These data demonstrate that only a subfraction of 15–20% of brain neuroblasts is missing due to cell death of NE progenitor cells in *EGFR^f2^* embryos.

Because during the period of neuroblast formation EGFR is active in the NE of ventral/intermediate DC and ventral TC, we analysed the number of ventral and intermediate neuroblasts in both neuromeres of *EGFR^f2^;Df(3L)H99* mutants. Ventral neuroblasts were reduced in TC and DC, whereas intermediate (Ind-positive) neuroblasts were all formed (figure 6*d*,*d′,e, e′,f*; electronic supplementary material, figure S5*e′,f′i*). Comparing the small number of neuroblasts missing in TC and DC with the total amount of brain neuroblasts missing in *EGFR^f2^* mutants (figure 6*c,f*; electronic supplementary material, figure S5*i*), we conclude that neuroblast formation is primarily affected in PC.

To ascertain if the failure in neuroblast formation in *EGFR^f2^* mutants is due to a deregulation of proneural genes, we investigated expression of *lethal of scute* (*l'sc*), the key proneural factor for the development of brain neuroblasts [47,48]. In *EGFR^f2^;Df(3 L)H99* mutants, *l'sc* expression is strongly reduced in regions where EGFR is normally active (figure 6*g–j*). Correspondingly, *l'sc* expression is significantly prolonged upon overexpression of activated Spi (*sca* *>* *sSpi*) (figure 6*k,l*). To test whether ectopic EGFR activation in those embryos is also sufficient to generate additional brain neuroblasts, we counted neuroblast numbers at stage 13 (until when *sca-Gal4* is broadly active; data not shown). The number of Dpn-positive brain neuroblasts was significantly increased (figure 6*m,n*), but not the mitotic index of those neuroblasts: stainings against the M-phase marker Phospho-Histone3 (PH3) showed that 23% of neuroblasts were mitotic in wild-type and 25% in *sca* *>* *sSpi* (*n* = 10 hemispheres each). Thus, EGFR activation is sufficient to induce proneural gene expression, and the formation of ectopic brain neuroblasts, but does not enhance their proliferative activity.

As EGFR is activated in the brain NE from stage 5 onwards, we finally asked if early EGFR signalling already impacts the extent of the NE by controlling the early mitotic activity (see [67]), and thus the final number of NE progenitor cells from which brain neuroblasts develop. Therefore, we analysed PH3-labellings with focus on the ventral/central head NE where EGFR is largely activated, and observed that the amount of mitotic NE cells is reduced by approximately 20% in *EGFR^f2^* mutants (*n* = 14 hemispheres) (electronic supplementary material, figure S5*j–m*). Nevertheless, this reduction in NE progenitor cell number alone does not account for the observed reduction in *l'sc* expression domains.

In sum, our data provide evidence that EGFR signalling affects formation of brain neuroblasts at multiple steps of development: first, by controlling mitosis in the NE anlagen to establish the proper number of neuroectodermal progenitor cells; second, by positively regulating expression of proneural gene *l'sc* in those cells; and third, by ensuring their survival (summarized in figure 6*o*).

## 3. Discussion

### 3.1. Localized EGFR signalling in the embryonic brain is controlled by neuromere-specific deployment of distinct ligands

**Figure 7.**
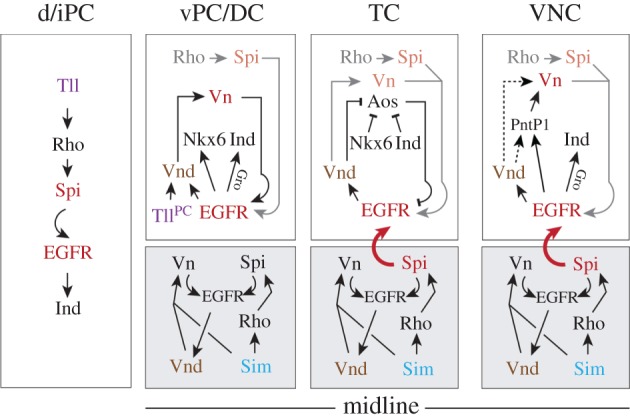
A model of genetic interactions between EGFR and other factors that pattern the brain in the DV axis, when compared with the VNC. Separated boxes show proposed genetic interactions in the NE (white boxes) of dorsal/intermediate (d/i) PC (left), ventral PC/DC (middle left), TC (middle right), VNC (right) and the ventrally adjacent mesectoderm (or midline; grey boxes), as deduced from the data in this study. In TC/DC, genetic factors/ligands active during the early period of EGFR signalling are indicated in grey/orange, respectively. Bent arrows indicate activity of secreted factors. Genetic control of EGFR is different in TC, DC and PC. For example, EGFR activity becomes midline-dependent only in TC (indicated by bent arrow in red), similar to the situation in the VNC. In the VNC, genetic interactions for PntP1 refer to [42]; it is unknown if EGFR signal induces *vn* expression via Vnd and PntP1 (indicated by stippled arrows). The potential role of PntP1 in the brain is unclear. See the main text for further details.

In the VNC, EGFR is activated in two phases. In the early, midline-independent phase, EGFR is induced by Rho (via processing of Spi) and Vn, which are both expressed in the ventral/intermediate NE; *rho* expression becomes restricted to the midline during gastrulation, while *vn* expression restricts towards the ventral NE [20,26,40,41]. In the following midline-dependent phase, EGFR activity essentially depends on Sim, a master regulator of midline development which induces *rho* expression (and Spi secretion) in the ventral midline and is required for *vn* expression in the ventral NE [35,64,65,68,69]. As summarized in figure 7, in the brain we observe a similar early period of EGFR activity before Sim expression is initiated in ventral mesectodermal cells (corresponding to the ventral midline; see also [43]). EGFR is initially induced by Rho and Vn expressed in the ventral/intermediate NE of TC, DC and ventral PC. However, only in the posterior brain, the TC, do both factors become confined towards the ‘ventral midline’, followed by a midline-dependent phase of EGFR signalling (figure 7). These findings support that patterning in the TC closely resembles the situation in the trunk, but is more derived in DC and PC where EGFR activity remains midline-independent, even though weakly Sim-positive midline cells ventral to DC are likely to secrete limited amounts of Spi and Vn. Our data show that in DC/PC, EGFR activity relies on neuroectodermal sources of Vn and Spi (processed by Rho) that are controlled by the DV gene *vnd* (as discussed below) and the terminal gap gene *tll*. Surprisingly, Vn, which plays only a minor role in VNC patterning [20,26,42,70], proved to be a major activating EGFR ligand in DC and ventral PC. The reason is probably the exceptionally low level of *rho* expression in this area, leading to low Spi-levels that alone cannot sufficiently activate EGFR. By contrast, Rho-dependent Spi is the only ligand in the largest part of PC (i.e. intermediate/dorsal PC). Despite a close spatial correlation, however, *rho* and MAPK signal levels correlate poorly in PC: a strong MAPK signal is detected in the anterior stripe despite very low *rho*-expression levels, whereas MAPK is weaker in the posterior stripe despite stronger *rho* expression levels. We noted that S expression levels correlate with MAPK activity, being strong in the anterior MAPK stripe, and weaker in the posterior MAPK stripe. S is responsible for transport of the Spi precursor to the Golgi, where Rho-dependent secretion of Spi occurs [30,31]. Rho also cleaves and inactivates S, thus compromising the levels of secreted Spi [71]. Therefore it seems likely that S together with Rho modulate localized levels of secreted Spi, and hence the activity of EGFR within distinct regions of the brain NE.

The nuclear Dorsal gradient is active in the early embryo to activate expression of *rho*, *vnd* and presumably *vn* in the truncal NE (each being expressed in a uniform longitudinal domain in the ventral NE) [9,72–74]. As the early expression domains of *rho*, *vnd* and *vn* cover in addition the ventral NE of the presumptive TC, DC and PC (figure 4*a,b*; electronic supplementary material, figure S4*a*), we assume that the early expression of these genes is likewise activated by the nuclear Dorsal gradient. In dorsal/intermediate PC, however, the induction of the slightly later emerging two stripes of *rho* expression might rather depend only on Tll (figure 7). The positive regulation of EGFR ligands by Tll in PC seems to be opposed to its function in the development of the larval visual system, where Tll has been proposed to block transcriptional programmes induced by EGFR (via Spi) [44].

Thus, during early brain development diverse ligands produced in distinct tissues (mesectoderm, NE) control localized activity of EGFR in a neuromere-specific manner (figure 7): in TC depending on midline-specific production of Vn and Spi (and Aos secreted from the dorsal TC, as discussed below), and in DC and PC primarily on Vn and Spi (via Rho), respectively, secreted from defined neuroectodermal domains. Other factors are known to control EGFR signalling, such as the feedback regulators Sprouty and Kekkon [75–78], MAPK phosphatase MKP-3 [79,80], or extracellular regulators of ligand travelling/activity such as Sulf1 and CG4096 [81]. Except Sulf1 and CG4096, which we did not detect in the embryonic brain NE (data not shown), the potential role of the other regulators in patterning of the embryonic brain has to be clarified in further investigations.

### 3.2. DV genes control activity of EGFR through regulation of *vn* and *aos*

In this study, we demonstrate a novel role for Vnd, Nkx6 and Ind in regulating the ligands of EGFR, which indicates that within the hierarchical gene network EGFR stands not strictly atop the DV genes (figure 7). The function of Vnd in this context is far-reaching, because Vnd induces *vn* expression in all brain neuromeres; this is of importance particularly in DC where Vn is the main EGFR ligand. In the VNC, *vn* expression is induced by the transcriptional activator Pointed P1 in response to EGFR signal [42] (figure 7). We cannot exclude that during posterior brain development Vnd induces *vn* expression by positively regulating Pointed P1.

Notably, Vnd also regulates the expression of the inhibitory ligand Aos in TC. Vnd suppresses *aos* expression in the ventral (and early intermediate) TC, and keeps Aos secretion limited to the dorsal TC (figure 7). Accordingly, Spi elicited from the ventral midline is able to activate EGFR only in ventral TC, where it maintains *vnd* expression. Vnd is also required for inducing expression of Nkx6 and Ind [52], which both keep *aos* suppressed in the intermediate TC after Vnd is downregulated.

Thus, via the deployment of EGFR ligands (Vn, Aos), Vnd acts positively on itself, and thus stabilizes at later stages the cross-repressive interactions between Msh (dorsal)/Nkx6 (intermediate) and Ind (intermediate)/Vnd (ventral), essential for establishing the boundaries of DV neuroectodermal and corresponding stem cell domains [52,53,82].

### 3.3. EGFR regulates expression of DV genes in the embryonic brain in a neuromere-specific manner

Subdivision of the NE into discrete gene expression domains is essential for the correct specification of neural stem cells. During DV patterning of the truncal NE, EGFR is necessary for regionalized expression of *vnd* and *ind* (summarized in [83]). In previous reports, we uncovered a network of genetic interactions underlying DV patterning in the brain (including *vnd*, *ind*, *msh*, *Nkx6*, *Ems*, *En*) [51–53]. Here, we expand on this knowledge and show that EGFR strongly participates in the control of DV gene expression in the early brain. EGFR signal is necessary for the maintenance of the expression of two *Nkx* genes, *Nkx6* and *vnd*, similar to its role for *vnd* expression in the VNC [17]. It is likely that EGFR signal regulates the regionalized expression of other patterning genes. For example, ectopic En is detected in a few NE cells in *EGFR^f2^*-mutant DC (figure 2*a,b*), making it likely that EGFR is involved in the control of *en* in this neuromere. As we previously showed that En negatively regulates the expression of *ind* [53], possibly the reduction of *ind* in the *EGFR^f2^*-mutant DC is partly due to ectopic En.

EGFR signal is necessary for *ind* activation in the trunk [17], whereas its effect on *ind* expression in the brain strongly differs between neuromeres (figure 7). In TC, *ind* expression is activated independently of EGFR, which is unique in the entire embryo. However, in *aos* mutants in which EGFR is ectopically activated in the intermediate TC (figure 4*l′′*), we observed a significant reduction of *ind* expression (data not shown). Thus, we propose that in TC, EGFR controls *ind* expression indirectly via the maintenance of the *ind*-repressor Vnd which in wild-type fades early in the intermediate TC to allow for *ind* expression.

In DC, *ind* activation requires both, EGFR signal and phosphorylation of the co-repressor Gro. Gro has been shown to be directly phosphorylated (and thereby inactivated) by EGFR/MAPK activity (and other RTK/MAPK pathways) [59], thereby regulating *ind* expression in the trunk [60]. Given that Ind is co-expressed with its Gro-dependent repressor Vnd [61], our results strongly suggest that EGFR allows *ind* expression in DC by phosphorylation of Gro, thus inactivating Vnd/Gro repressor complexes. We noted that in the ventral NE of the trunk and TC, unlike in DC, Vnd manages to repress *ind* expression despite EGFR being active and Gro being phosphorylated. As Vnd has been shown to form multiple complexes in the embryo [84], a possible explanation is that Vnd associates with RTK-insensitive co-repressors in trunk segments, and exclusively with Gro in DC (thus being sensitive to Gro inactivation). In PC, EGFR is also necessary for *ind* activation, but not sensitive to Gro-phosphorylation (data not shown), indicating an EGFR-dependent regulatory mechanism different from VNC and other brain neuromeres.

### 3.4. EGFR controls the formation of brain neuroblasts at different developmental steps

About 40% of brain neuroblasts are missing in *EGFR^f2^*-mutant embryos at embryonic stage 11. Our data suggest that EGFR function affects the formation of brain neuroblasts at multiple steps of development. First, activated EGFR positively controls the early mitotic activity within the neuroectodermal anlagen, in accordance with EGFR function in other developmental contexts (e.g. [85–88]). Second, as many neuroectodermal progenitor cells undergo premature cell death in *EGFR^f2^* mutants, EGFR signalling is critical for their survival (see also [43,45]). EGFR-dependent survival has been reported also for midline glial cells in which Spi-activated EGFR suppresses the proapoptotic protein Hid [89,90]. Also in the large dorsal/intermediate PC, where apoptosis of *EGFR^f2^*-mutant neuroectodermal cells is substantial, Spi seems to be the only EGFR ligand, raising the possibility that a similar mechanism regulates neuroectodermal cell survival. We identified a small number of brain neuroblasts with activated EGFR, but never apoptotic neuroblasts in *EGFR^f2^* mutants, suggesting that EGFR signal is rather dispensable for their survival. Third, in addition to the decrease of neuroectodermal progenitor cells, impairment of neuroblast formation in all brain neuromeres of *EGFR^f2^* mutants is due to the loss of proneural gene expression; *l'sc* needs EGFR signal to be properly activated, in compliance with findings in the larval optic lobe [91]. In accordance with the distribution of activated EGFR in wild-type, in cell-death-deficient *EGFR^f2^;Df(3L)H99* mutants, we found a large population of neuroblasts to be missing at all DV positions in PC, and specifically ventral neuroblasts in the posterior brain (TC/DC). By contrast, in the *EGFR*-mutant VNC intermediate neuroblasts do not develop, whereas ventral neuroblasts usually form but are often misspecified [20]. In VNC, EGFR promotes the formation of intermediate neuroblasts by activating *ind* expression [17,20]. At least in PC, it is likely that activity of Ind, in addition to L'sc, both of which are strongly reduced in *EGFR^f2^* mutants, control the development of a small subset of protocerebral neuroblasts. Even though EGFR is active, in DC it does not impact the formation of intermediate neuroblasts; as these neuroblasts, opposed to the NE, express *ind* in *EGFR^f2^* mutants, this further suggests that EGFR activity is dispensable for *ind* expression in these progenitors. In TC, onset of *ind* expression is delayed and regulated independently of EGFR (in NE and neuroblasts), explaining that intermediate neuroblasts are unaffected in the *EGFR^f2^*-mutant TC. However, in the mutant TC, we recognized an almost entire loss of ventral neuroblasts, which develop late. Moreover, L'sc was largely lacking there, and Vnd dissipated in the NE before these neuroblasts normally develop. Thus, it is likely that Vnd (see also [92]), together with L'sc, promote formation of these late-developing ventral neuroblasts. As Vnd is still expressed in the remaining ventral brain neuroblasts in *EGFR^f2^* mutants (electronic supplementary material, figure S5*f′*), this suggests that *vnd* (similar to *ind*) expression is differently regulated in NE and neuroblasts (see also [24]), and further, that these ventral brain neuroblasts do not undergo a fate shift towards intermediate identity, as has been observed for approximately 50% of ventral neuroblasts in the *EGFR*-mutant VNC [20].

### 3.5. Phylogenetic considerations of EGFR-regulated patterning in the brain

The key components of the EGFR signalling pathways are evolutionarily highly conserved from fly to human. In vertebrates, 4 EGFR family members (ErbB1-4, with ErbB1 homologous to EGFR) and 11 EGF-like ligands are known (reviewed in [93,94]). In the forebrain, ErbB ligands secreted from a narrow region between the dorsal and ventral telencephalon (called ‘antihem’) have been proposed to assist in maintaining DV fates, which suggests a possible involvement of EGFR signalling in regional patterning of the cerebral cortex [95]. ErbB signalling might also be involved in patterning and differentiation of structures at the midbrain–hindbrain boundary (reviewed in [96]). However, ErbB signalling has not been connected with regulation of DV patterning genes (i.e. *vnd/Nkx2*, *Nkx6*, *ind/Gsh*). Instead, several other extrinsic signalling molecules are involved in their regulation, including the key player Shh, which is secreted from the floorplate (reviewed in [97–99]). This suggests that different upstream signalling pathways are used to control the expression of DV patterning genes in insect and vertebrate brains, even though the regionalized expression of these genes exhibits certain similarities in the embryonic brain of both animal phyla [52,100].

## 4. Material and methods

### 4.1. *Drosophila* genotypes

The following fly strains were used: Oregon R (wild-type); *aos^Δ7^* [37], *Df(3L)H99* [66], *EGFR^f2^* [101], UAS-*sim* [102]; UAS-*sSpi* [21]; *Matα*-Gal4 [55], *NGT40*-Gal4 [62], *sim^H9^* [64], *tll^l49^* [103] (all provided by Bloomington Drosophila Stock Center); *rho^PΔ5^* [37] (provided by Marta Llimargas Casanova); *sca*-Gal4 [57] (provided by Uwe Hinz); UAS-*Gro^AA^*, UAS-*Gro^DD^* [58] (provided by Ze'ev Paroush); UAS-*tll* [104] (provided by Mitsuhiko Kurusu); *vn^RG436^* [105] (provided by Amanda Simcox); UAS-*vnd* [13], *vnd^6^* [6] (provided by James Skeath).

### 4.2. Staging, flat preparation and mounting of embryos

Flat preparations of the head/truncal ectoderm of stained embryos and mounting were carried out as described previously [106].

### 4.3. Immunohistochemistry

Embryos were dechorionated, fixed and immunostained according to previously published protocols [48]. The following primary antibodies were used: mouse-anti-Dachshund 2–3 (1 : 250) [107], mouse-anti-Invected 4D9 (1 : 7) [108], mouse-anti-Prospero (1 : 10) (all provided by DSHB); rabbit-anti-Death caspase-1 (#9578) (1 : 50), rabbit-anti-p44/42-MAPK (1 : 500) (both provided by Cell Signalling Technology); guinea pig-anti-Deadpan (1 : 5000) [109] (provided by Jürgen Knoblich); rabbit-anti-Engrailed (1 : 800) (Santa Cruz Biotechnology); rabbit-anti-Ind (1 : 1000) [110] (provided by Tonia von Ohlen); mouse-anti-p44/42-MAPK (1 : 2000) (provided by Sigma Aldrich); rabbit-anti-PH3 (1 : 500) (provided by Merck Millipore); guinea pig-anti-Runt (1 : 300) [111] (provided by Ralf Pflanz); guinea pig-anti-Sim (1 : 1500) [112] (provided by Stephen Crews); rabbit-anti-Vnd (1 : 2000) [16] (provided by Marshall Nirenberg); sheep-anti-DIG alkaline-phosphatase conjugated (1 : 1000) (provided by Roche Diagnostics). The secondary antibodies were either biotinylated, conjugated with alkaline-phosphatase, or DyLight, Cyanine (all Jackson Immunoresearch) and Alexa (Life technologies) fluorescent dyes (all diluted 1 : 500). Tyramide signal amplification (TSA biotin system; PerkinElmer) was used in DAB stainings according to the manufacturer's protocol.

### 4.4. Whole mount *in situ* hybridization

Probes were synthesized using either linearized cDNA/EST-clones, cloned PCR products or PCR products containing a RNA polymerase adapter (both from genomic DNA) (electronic supplementary material, table S1) as a template. T3, T7 or SP6 Polymerase and DIG-RNA Labelling Mix (all Roche Diagnostics) were used for probe synthesis according to the manufacturers protocol. *In situ* hybridization was performed as described previously [51] and the probes processed with NBT/BCIP or VectorRed (Vector Labs) solution. Afterwards, the embryos were immunolabelled with a second primary antibody followed by incubation with biotinylated secondary antibodies, and processed with DAB.

### 4.5. Documentation

The non-fluorescent stainings were documented on a Zeiss Axioplan. Pictures were digitized with a CCD camera (Contron progress 3012). Fluorescent confocal images were acquired on a Leica TCS SP5 II. Pictures were processed with ImageJ, Adobe Photoshop CS4 and Adobe Illustrator CS4. Data (shown in figures 2*p* and 6*c,f*; electronic supplementary material, figure S5*i,m*) were analysed with a two-tailed unpaired Student's *t*-test.

## Supplementary Material

Genetic regulation and function of EGFR signalling in patterning of the embryonic Drosophila brain by Urbach et al - Supplemental Material
