# Supplementary material for: Genetic regulation and function of epidermal growth factor receptor signalling in patterning of the embryonic Drosophila brain
Source: Open Biol. 2016 Dec 14;6(12):160202. doi: 10.1098/rsob.160202 (PMC5204121; doi:10.1098/rsob.160202)
Supplement: Genetic regulation and function of EGFR signalling in patterning of the embryonic Drosophila brain by Urbach et al - Supplemental Material [file rsob160202supp1.pdf]

Supplemental Material

Supplemental Material and Methods

Probe	Template (Source)	Vector	Restriction enzyme	Poly-merase	5'-primer (5'-3')	3'-primer (5'-3')*
l'sc	EST-clone RE59335 (DGRC)	pFLC1	PstI	T3	-	-
ind	cDNA (E.Bier)	pBS SKII(+)	XhoI	T7	-	-
msh	cDNA (E.Bier)	pBS SKII(+)	HindIII	T7	-	-
vnd	cDNA (E.Bier)	pBS SKII(+)	SacI	T7	-	-
Nkx6	EST-clone RE18506 (DGRC)	pFLC1	SphI	T3	-	-
rho	EST-clone LD06131 (DGRC)	pBS SKII(-)	SwaI	T3	-	-
S	EST-clone AT04225 (DGRC)	pOTB7	BclI	T7	-	-
vn	EST-clone IP15007 (DGRC)	pOT2	BglII	T7	-	-
tll	EST-clone IP01133 (DGRC)	pOT2	HindIII	T7	-	-
aos	PCR-product	-	-	SP6	GACGAGGTCAACATTAACTC	ATTTAGGTGACACTATAGAAGAGTTTGACGTTTGCTGCGTTGC

Probes were synthesized from linearized plasmids containing cDNA (*ind*, *msh*, *vnd*) or EST clones (*l'sc, Nkx6, rho, S, vn, tll*), or using a purified PCR product containing a SP6 RNA polymerase promoter (underlined sequence) reverse complementarily attached 3' to the amplified exon sequence via the 3'primer (*aos*). T3, T7 or SP6 Polymerase and DIG-RNA Labeling Mix (all Roche Diagnostics) were used for probe synthesis according to the manufacturers protocol.

Supplemental Figures

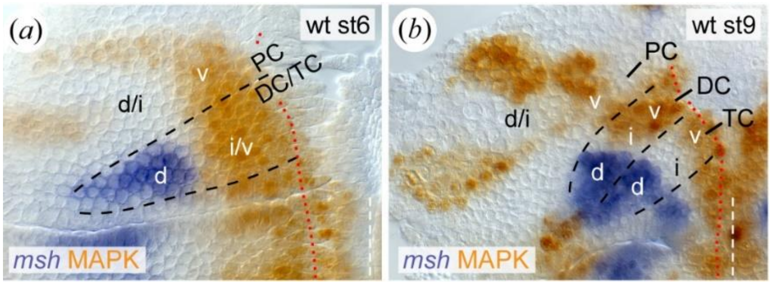

Figure S1. Pattern of MAPK in relation to *msh* expression.
(***a,b***) Flat preparations displaying the head ectoderm of the left hemisphere; anterior is up. MAPK pattern, representing EGFR activity, combined with *msh* expression. The pattern of MAPK remains complementarily to *msh* expression from stage (st) 6 (*a*) to stage 9 (*b*). Black dashed lines indicate borders between tritocerebrum (TC), deutocerebrum (DC) and protocerebrum (PC). White dashed lines indicate ventral midline. Red dotted lines indicate the border between neuroectoderm and mesectoderm. d,i,v, dorsal /intermediate/ventral neuroectoderm, respectively.

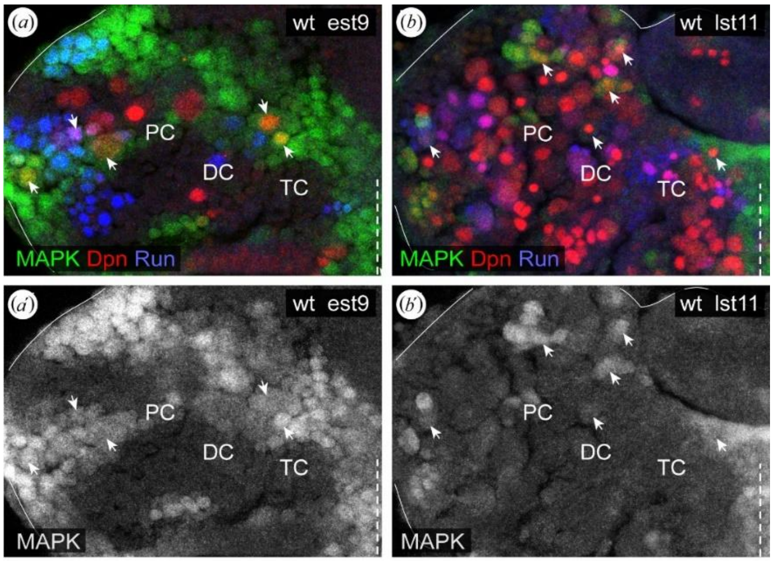

Figure S2. EGFR activity in brain neuroblasts.
(***a-b´***) MAPK pattern combined with the pan-neuroblast marker Deadpan (Dpn) and the neuroblast-subset marker Runt (Run) (Urbach and Technau, 2003b) in a wildtype hemisphere at early stage 9 (est9; *a,a´*) and late stage 11 (lst11; *b,b'*). (*a,a'*) By position and expression of Runt about five, transiently MAPK-positive neuroblasts are identified at stage 9 (arrowheads) in the deutocerebrum (DC) and protocerebrum (PC). (*b,b´*) A different set of about 5-6 MAPK-positive neuroblasts (arrowheads) is detected at late stage 11. For orientation, and other abbreviations and symbols see figure S1.

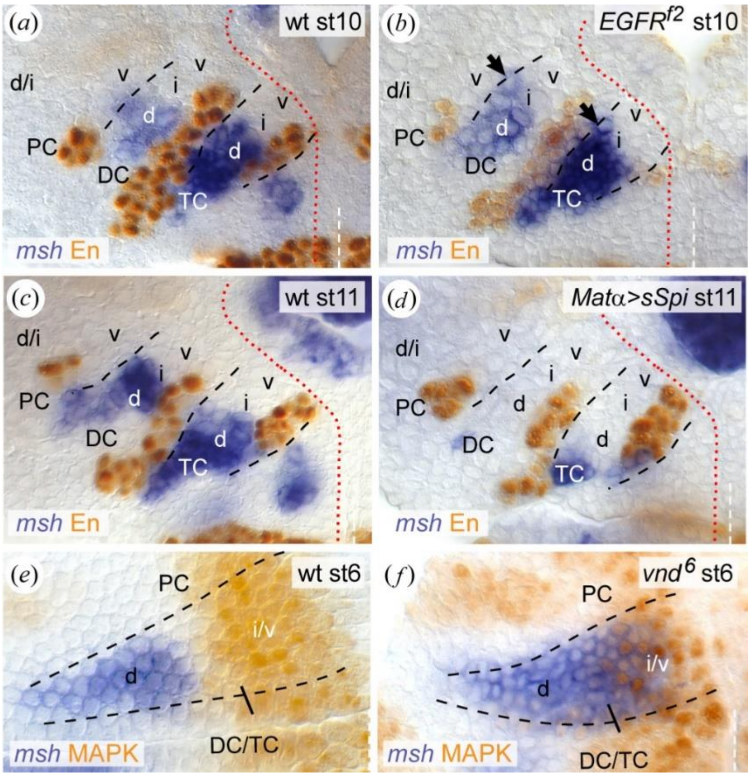

Figure S3. EGFR controls expression of *msh*.
(***a-d***) Double stainings against *msh* and En. As compared to wild-type (wt) (*a*), at stage 10, *msh* expression is slightly derepressed in intermediate TC and DC (arrow) in *EGFR*^{f2} mutants (48% of TC, and 56% of DC; n=27 each) (*b*). Conversely, *msh* is repressed in TC and DC at stage 11 upon *Matα>sSpi* (76% of TC and DC; n=48) (*d*), compared to wild-type (*c*). (***e,f***) Double stainings against *msh* and MAPK. (*e*) *msh* is expressed complementary to MAPK in wild-type at stage 6. (*f*) In *vnd*^{6} mutants, *msh* expression expands into intermediate/ventral TC and DC. As under these conditions *msh* expression is partly co-detected with MAPK, this demonstrates that activated EGFR does not directly repress *msh*. For orientation, and other abbreviations and symbols see figure S1.

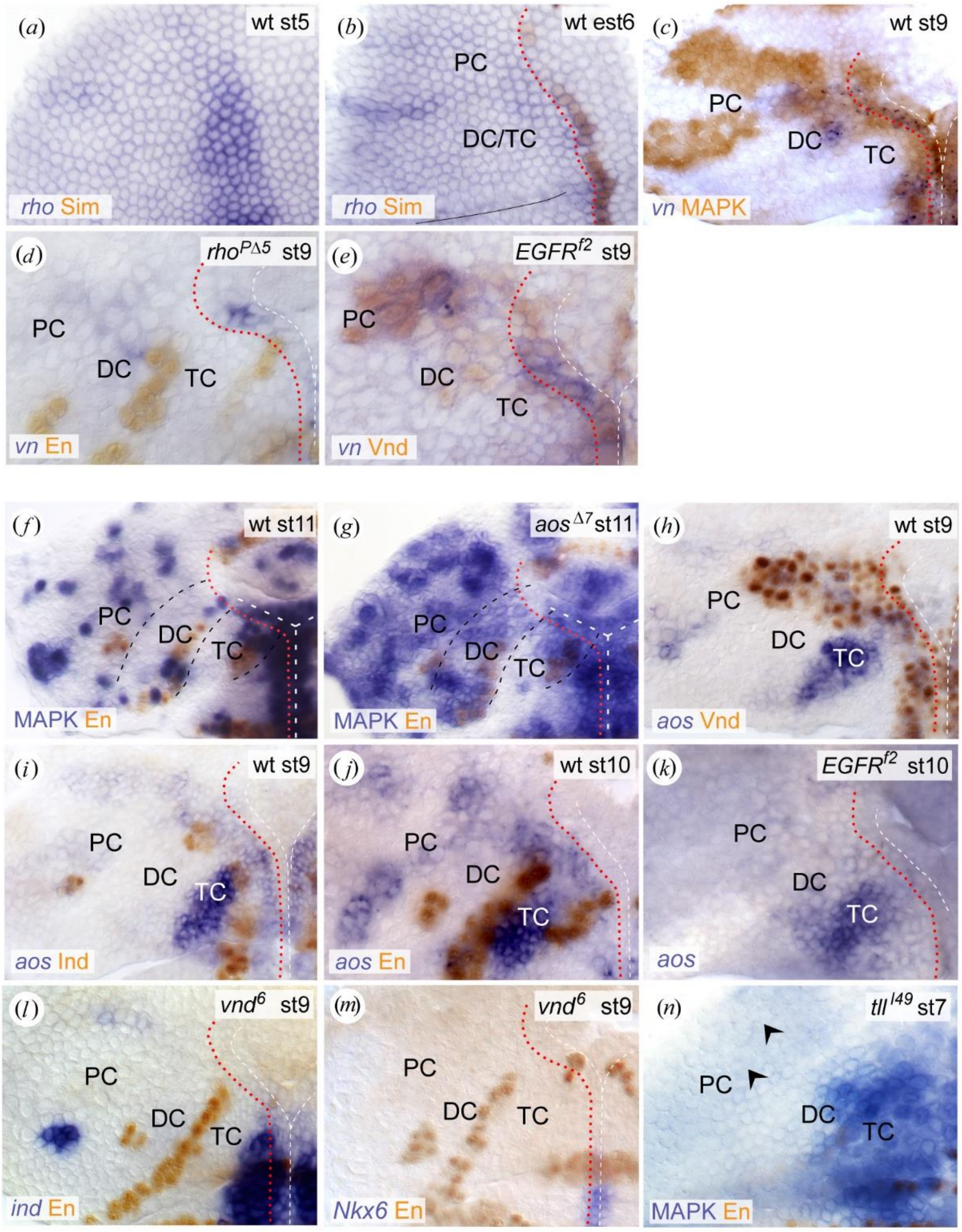

Figure S4. Vnd controls expression of *vn*, and Ind/Nkx6 control expression of *aos*.
(***a,b***) Double stainings against *rho* and Sim in wild-type (wt) at stage (st) 5 (*a*) and late stage 6 (*b*). *rho* expression is initiated independently of Sim in the ventral neuroectoderm at stage 5 (*a*), and restricts towards the Sim-expressing midline during stage 6 (*b*). (***c***) *vn*/MAPK double staining in wild-type at stage 9. *vn* expression correlates with EGFR activity in the brain neuroectoderm, except in intermediate/dorsal PC. (***d***) *vn*/En double staining in a $rho^{P\Delta 5}$mutant at stage 9. *vn* expression is reduced. (***e***) *vn*/Vnd expression in an $EGFR^{f2}$ mutant at stage 9. Reduced *vn* expression is detected only in the Vnd-positive neuroectoderm. (***f,g***) MAPK/En staining in wildtype (*f*) and $aos^{\Delta 7}$ mutants (*g*). MAPK is endogenously largely downregulated in the wildtypic brain NE at stage 11 (*f*), whereas MAPK activity is prolonged and expanded in $aos^{\Delta 7}$ mutants (compared to earlier MAPK activity in figure 1). (***h,i***) *aos*/Vnd (*h*) or *aos*/Ind (*i*) double stainings in wild-type at stage 9 show that *aos* expression is confined to dorsal TC, i.e. dorsal to the tritocerebral Vnd and Ind domain. (***j,k***) *aos*/En double staining in wild-type (*j*), and *aos* staining in an $EGFR^{f2}$ mutant (*k*) at stage 10. *aos* expression is eliminated in $EGFR^{f2}$ mutants, except in dorsal TC (*k*). This demonstrates that *aos* is activated independently of EGFR signaling in dorsal TC. (***l,m***) Expression of *ind* (*l*) or *Nkx6* (*m*) combined with

En in *vnd*^{6} mutants at stage 9. *ind* and *Nkx6* are lost in TC and DC. (***n***) MAPK/En expression in a *tll*^{l49} mutant. MAPK is missing in intermediate/dorsal PC (arrowheads) already at stage 7. For orientation, and other abbreviations and symbols see figure S1.

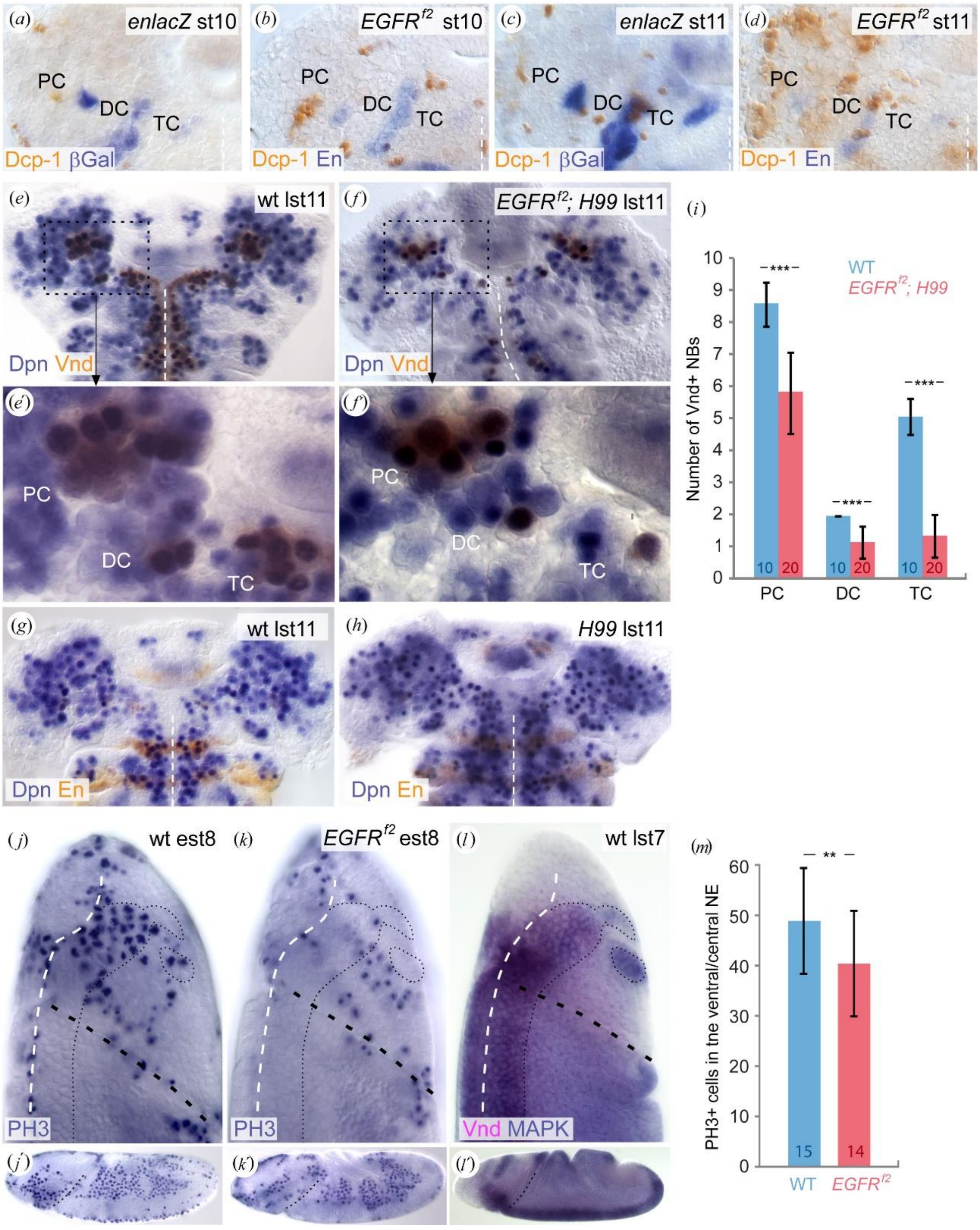

FigureS5. EGFR activity controls early proliferation and survival of the neuroectoderm, and formation of neuroblasts in the embryonic brain.

(*a-d*) D-Casp1-labeling combined with *en-lacZ* (*a*,*c*) or En (*b*,*d*) at stages (st) 10 and 11. In the brain neuroectoderm, cell death starts at stage 10 in wild-type (*a*) and EGFR^{f2} mutants (*b*), but proceeds until stage 11 significantly stronger in EGFR^{f2} mutants (*d*) than in wild-type (*c*). (***e-f´***) Vnd/Dpn double staining at late stage 11. Vnd/Dpn-positive neuroblasts are missing in TC, DC and PC of *EGFR^{f2}; H99* mutants (*f*), compared to wild-type (*e*). (*e´*,*f´*) Higher magnifications of areas boxed in (*e*,*f*). Note that at stage 11 only a subset of ventral neuroblasts in DC is Vnd-positive. Accordingly, in *EGFR^{f2}; H99*-mutant DC, the number of ventral neuroblasts missing is higher (compare with figure 6*h*). (***g***,***h***) Dpn/En stainings in wild-type (*g*) and *Df(3L)H99* (*h*) at late stage 11. The number of Dpn-positive neuroblasts is not affected in *Df(3L)H99* embryos. (***i***) Loss of Vnd-positive neuroblasts in TC, DC and PC is significant; numbers within bars indicate n, error bars indicate s.d.; ***p<0.0001. (***j-l´***) Whole mount embryos stained against PH3 at early stage 8 (*j-k´*), and against Vnd/MAPK at late stage

7 (l,l´), in a ventrolateral (*j*,*k*,*l*) and lateral view (*j´*,*k´*,*l´*). Bold black dashed lines indicate the cephalic furrow, white dashed lines indicate the ventral midline. The fine black dashed lines delineate the MAPK domains in the ventral and central brain neuroectoderm in (*j*,*k*), according to (*l*). (Number of PH3-positive cells is significantly reduced in the ventral/central brain neuroectoderm in $EGFR^{f2}$ mutants*k*, as compared to wild-type (*j*), which is evaluated in (***m***); **p<0.01.

Supplemental References

Urbach, R., Technau, G.M. (2003a). Molecular markers for identified neuroblasts in the developing brain of Drosophila. *Development* **130**, 3621-3637.
